# Neurotransmitter identity and electrophysiological phenotype are genetically coupled in midbrain dopaminergic neurons

**DOI:** 10.1038/s41598-018-31765-z

**Published:** 2018-09-11

**Authors:** Mónica Tapia, Pierre Baudot, Christine Formisano-Tréziny, Martial A. Dufour, Simone Temporal, Manon Lasserre, Béatrice Marquèze-Pouey, Jean Gabert, Kazuto Kobayashi, Jean-Marc Goaillard

**Affiliations:** 1 Unité de Neurobiologie des Canaux Ioniques et de la Synapse, INSERM UMR 1072, Aix Marseille Université, 13015 Marseille, France; 2 Département de Biochimie et Biologie Moléculaire, Hôpital Nord, Marseille, France; 3 Department of Molecular Genetics, Institute of Biomedical Sciences, Fukushima Medical University, Fukushima, 960-1295 Japan

## Abstract

Most neuronal types have a well-identified electrical phenotype. It is now admitted that a same phenotype can be produced using multiple biophysical solutions defined by ion channel expression levels. This argues that systems-level approaches are necessary to understand electrical phenotype genesis and stability. Midbrain dopaminergic (DA) neurons, although quite heterogeneous, exhibit a characteristic electrical phenotype. However, the quantitative genetic principles underlying this conserved phenotype remain unknown. Here we investigated the quantitative relationships between ion channels’ gene expression levels in midbrain DA neurons using single-cell microfluidic qPCR. Using multivariate mutual information analysis to decipher high-dimensional statistical dependences, we unravel co-varying gene modules that link neurotransmitter identity and electrical phenotype. We also identify new segregating gene modules underlying the diversity of this neuronal population. We propose that the newly identified genetic coupling between neurotransmitter identity and ion channels may play a homeostatic role in maintaining the electrophysiological phenotype of midbrain DA neurons.

## Introduction

Most neuronal types have a well-defined electrophysiological phenotype that they reliably establish and maintain over their (sometimes very long) lifetime. The electrophysiological phenotype is defined by the types of ion channels expressed by the neuron, their subcellular location and their interaction with the neuron’s passive properties^1,2^. Moreover, theoretical and experimental studies have demonstrated that precise levels of ion conductances are critical to define a given pattern of activity^3–6^. Paradoxically, the levels of expression of many ion channels have been shown to exhibit several-fold cell-to-cell variability in a same neuronal type^7–11^. This might be explained by ion channel degeneracy^12^, whereby variability at a single ion channel level is compensated by variations in functionally overlapping ion channels^3,13–16^. Indeed, several studies in invertebrates have shown that ion channel expression levels can be correlated in a cell type-specific manner^9–11^. Therefore, a complete understanding of the genesis and stability of electrical phenotype can only be achieved using systems-level approaches simultaneously investigating the levels of expression of most of the ion channels expressed by a given neuronal type.

Midbrain DA neurons of the ventral tegmental area (VTA) and substantia nigra pars compacta (SNc) *in vitro* display a characteristic low-frequency pacemaking activity, a broad action potential and a hyperpolarization-induced sag. Although these properties exhibit significant cell-to-cell quantitative variations^5,17,18^, the combination of these features represents a qualitative fingerprint making midbrain DA neurons immediately distinguishable from their neighboring GABAergic neurons of the substantia nigra pars reticulata (SNr)^19,20^. How DA neurons acquire this specific electrophysiological signature and maintain it is a question that still awaits a complete answer. As these neurons are spontaneously active in the absence of synaptic inputs, much emphasis has been put on the study of their voltage-gated conductances, and many ion channel types have been identified at the mRNA, protein and/or functional levels^21–23^. For instance the Cav1.3 calcium channels and sodium channels have been shown to be involved in generating the subthreshold oscillations driving the spontaneous firing of these neurons^4,24–26^. Hyperpolarization-activated ion channels (in particular HCN2 and HCN4) positively modulate pacemaking frequency^17,27,28^ while the Kv4.3 potassium channels negatively regulate this same activity^7^. Finally, small-conductance calcium-activated potassium channels, SK2 and SK3 mainly, control the regularity of pacemaking^18,29,30^. However, simultaneous quantitative measurements of the levels of expression of these different ion channels are still missing.

In the present study, we investigated the levels of expression of several voltage-gated ion channels in midbrain DA neurons using single-cell reverse transcription quantitative PCR (sc-RTqPCR). Other genes, related to neurotransmitter metabolism, calcium signaling and neuronal structure, were also investigated. Using multivariate mutual information analysis (I_k_ analysis) designed to decipher high-dimensional statistical dependences in datasets, we found that the expression levels of several ion channels were genetically coupled with DA metabolism genes, unravelling a co-regulatory module linking neurotransmitter identity and electrophysiological phenotype. Consistent with previous studies, other genes (including ion channels) displayed significant heterogeneity in their expression pattern. However, the newly identified genetic coupling was found in all midbrain DA neurons, suggesting that the rules underlying the definition and stability of their electrophysiological phenotype are conserved.

## Results

**Figure 1:**
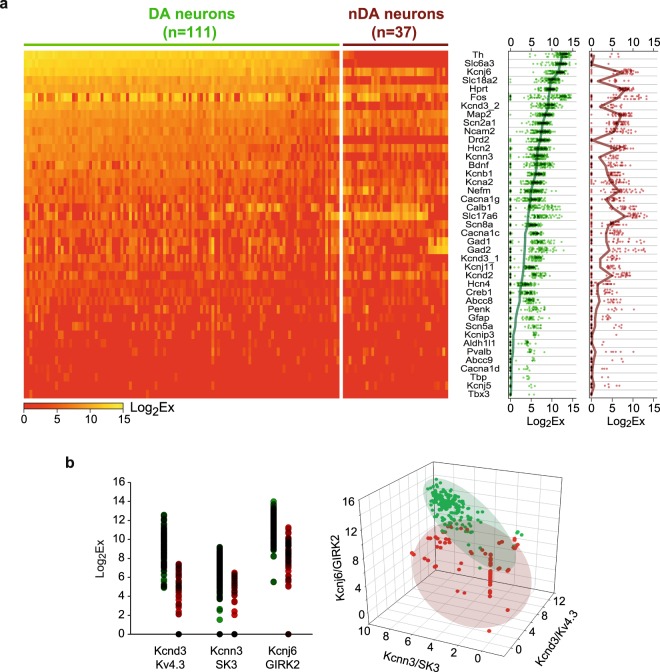
Cell-to-cell variation in gene expression levels in midbrain DA and nDA neurons. (**a**) Levels of expression (Log_2_Ex) of 41 genes in the collected 111 DA and 37 nDA neurons represented as a heatmap (left) or as a scatter plot (right). The thick green and red lines in the scatter plot represent the average expression levels while each dot corresponds to the expression level in one neuron. Neurons in the heatmap are ordered based on Th and Slc6a3 levels of expression in DA neurons (thick green line) and genes are ordered based on their average level of expression in DA neurons (left plot). (**b**) Levels of expression (Log_2_Ex) of the ion channels *Kcnd3*/Kv4.3, *Kcnn3*/SK3 and *Kcnj6*/GIRK2 in DA (green) and nDA neurons (red) represented as a one-dimensional plot (left) or 3-dimensional plot (right). Shaded ellipses outline the global distribution of the data points in the 3-dimensional space.

In order to obtain precise measurements of cell-to-cell variability in gene expression^31^, we performed sc-RTqPCR on acutely dissociated midbrain DA neurons. TH-GFP mice were used to identify putative DA neurons (Supplementary Fig. 1a), which displayed the expected electrophysiological properties^32,33^ (Supplementary Fig. 2). DA and nDA phenotypes were confirmed and refined based on the combined expression of *Th*/TH and *Slc6a3*/DAT (DA transporter) or lack thereof ^34^, allowing neurons collected from wild-type animals to be included (Supplementary Fig. 1b). Based on *Th*-*Slc6a3* expression, 111 neurons were classified as DA and 37 as nDA, the latter henceforth being considered merely as a negative control in our analysis. We quantified the levels of expression of 41 genes (Fig. 1a), including 19 related to ion channel function and 9 related to neurotransmitter definition (see Supplementary Fig. 1c and Supplementary Table 1).

Most genes displayed significant variability in their expression levels (including dropout events) across cells (Fig. 1a, Supplementary Fig. 3). As expected, DA neurons expressed high levels of DA metabolism and signaling-related genes such as *Th*/TH, *Slc6a3*/DAT, *Slc*1*8a2*/VMAT2 and *Drd2*/D2R. Midbrain DA neurons also displayed lower and variable levels of *Slc*1*7a6*/VGLUT2, *Gad1*/GAD67 and *Gad2*/GAD65, consistent with their documented heterogeneity^18^. While some ion channels showed similar expression profiles in DA and nDA neurons (i.e *Cacna1c*/Cav1.2, *Cacna1g*/Cav3.1 and *Hcn2*/HCN2), others (*Kcnb1*/Kv2.1, *Kcnd3_2*/Kv4.3 and *Kcnj6*/GIRK2) displayed higher levels of expression in DA neurons. Interestingly, although the average expression profiles were different between DA and nDA neurons, there was considerable overlap in the expression levels due to the high degree of cell-to-cell variability in expression (Fig. 1a,b, Supplementary Fig. 3). Interestingly, DA and nDA neurons could be separated when represented in a 3-dimensional space defined by the levels of expression of genes with substantial gene expression overlap (for instance *Kcnd3*/Kv4.3, *Kcnn3*/SK3 and *Kcnj6*/GIRK2, see Fig. 1b).

### Cell type-specific patterns of correlations in gene expression levels in midbrain DA neurons

**Figure 2:**
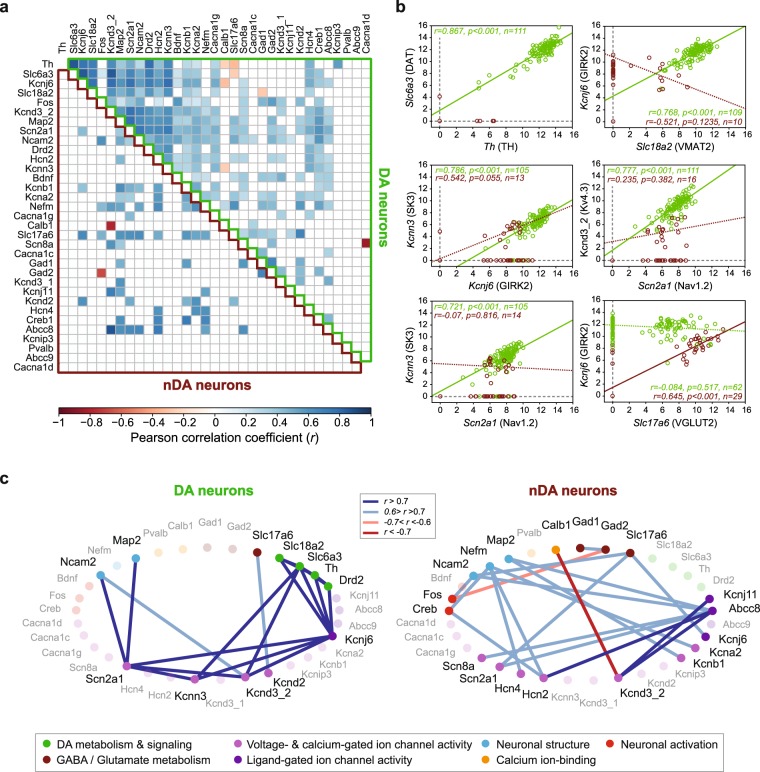
Second order linear analysis reveals specific patterns of correlations in gene expression levels in midbrain DA and nDA neurons. (**a**) Heatmap representing the significant correlations in expression levels for 33 genes in DA neurons (upper right triangle) and nDA neurons (lower left triangle) (Pearson correlation coefficient). Correlations were processed on non-zero values of expression, and only correlations with a p value < 0.05 and n > 5 are represented. Please note the difference in patterns of correlations between DA and nDA neurons.(**b**) Scatter plots presenting examples of significant correlations in gene expression levels. Green and dark red dots correspond to DA and nDA neurons, respectively. r, p, and n values are displayed for each correlation test. Plain and dotted lines indicate significant and non-significant Pearson correlations, respectively. Protein names are given in parentheses. (**c**) Scaffold representations of the 20 most significant correlations in expression levels in DA (left) and nDA (right) neurons (r values > 0.6 or < −0.6, see color coding in the top middle box). Genes were ordered based on the known function of the corresponding proteins (see table at the bottom for the color-coding of functions). Genes involved in the depicted correlations are highlighted (dark font). Please note the strong connectivity between DA metabolism/signaling and ion channel genes in DA neurons.

Previous studies have suggested that cell-to-cell variability at the single gene level might hide stable cell type-specific higher-order relationships, such as correlations in the levels of expression of genes^9,35^. Notably, strong correlations in ion channel expression levels were found in 7 distinct neuronal types in the crustacean nervous system^9,10^. As a first step, we performed Pearson correlation analysis on the 33 most relevant genes (Fig. 2, Supplementary Fig. 4). The patterns of correlations were found to be very different between the DA and nDA neurons, even for genes that displayed similar levels of expression in both cell types: *Kcnj6*/GIRK2 *vs Scn2a1*/Nav1.2 for instance in DA neurons, *Scn2a1*/Nav1.2 *vs Slc17a6*/VGLUT2 in nDA neurons (Fig. 2; Supplementary Fig. 4). Interestingly, several ion channels were involved in these cell type-specific correlations (*Scn2a1*/Nav1.2, *Kcnn3*/SK3, *Kcnd3*/Kv4.3, *Kcnj6*/GIRK2, Fig. 2b). However, the most significant observation was that some of the strongest correlations found in DA neurons linked the group of genes involved in DA metabolism and signaling (*Th*/TH, *Slc6a3*/DAT, *Slc18a2*/VMAT2, *Drd2*/D2R) to a group of ion channels (*Kcnj6*/GIRK2, *Kcnd3_2*/Kv4.3, *Kcnn3*/SK3, *Scn2a1*/Nav1.2), suggesting the existence of a large module of co-regulated genes relating neurotransmitter identity to electrical phenotype (Fig. 2c).

### Multivariate mutual information analysis framework

**Figure 3:**
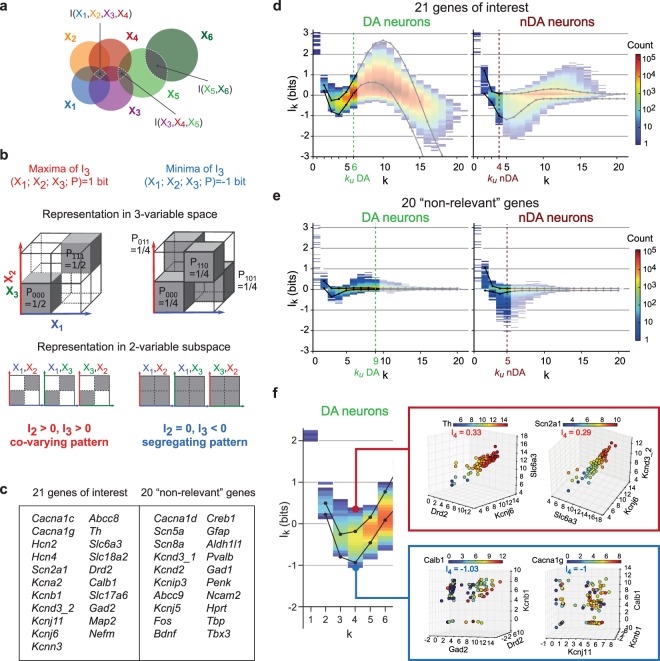
High order analysis identifies gene modules with co-varying and segregating patterns in DA neurons. (**a**) Venn diagram illustrating a system of 6 random variables sharing mutual information at degree 2, 3 and 4. (**b**) Theoretical examples of positive (left) and negative (right) I_3_ for 3 binary random variables X_1_, X_2_, X_3_ represented as 3- and 2-variable spaces. Note that the segregating pattern of negativity is only visible in 3-dimensional space (complete explanation in Supplementary Fig. 5). (**c**) List of the 21 genes of interest (left) used for mutual information analysis and the remaining 20 genes considered as “non-relevant” (right). Note that the term “non-relevant” is used only in the context of mutual information analysis to distinguish between more or less informative genes. (**d**) Information landscape for the 21 genes of interest for DA (left) and nDA neurons (right). The undersampling limit or k_u_ for DA and nDA neurons is represented on every landscape as a vertical dotted line and increased transparency of the graph. The upper and lower statistical limits, the top or bottom 5% fixed by the averaged shuffled landscape, are represented on the graphs as black lines. (**e**) Information landscapes for the 20 other “non-relevant” genes. (**f**) Magnification of the landscape presented in (**d**) for DA neurons until dimension k = 6. The 4D-scatter plots in the colored insets on the right represent the levels of expression of 2 quadruplets of genes sharing strong positive I_4_ (upper plots) and strong negative I_4_ (bottom plots) in DA neurons.

While pairwise analysis has been useful to provide insights about gene regulatory relationships^9,35,36^ it is conceivable that the complex architecture of gene regulation might manifest in higher-order, or non-linear relationships. Various information theory approaches have been proposed to define gene regulatory modules, based on the exploration of higher-order relationships, notably three-way interactions^37,38^. However, genetic regulation might involve interactions of even higher-order^39^. Based on the information co-homology formalized by Baudot and Bennequin^40^, we recently developed an analysis that combines, in a single framework, statistical and topological analysis of gene expression for systematic identification and quantification of higher-order interactions in gene expression^41^. In this framework, joint-entropy (H_k_) and multivariate mutual information (I_k_) quantify the variability/randomness and the statistical dependences of the variables, respectively (Fig. 3a, see Methods). H_k_ and I_k_ are defined as follows^40,42,43^:

$${H}_{k}=H({X}_{1},\,{X}_{2},\,\mathrm{...},\,{X}_{k})\,=H({X}_{I})$$

$${I}_{k}({X}_{1};\,\mathrm{...};\,{X}_{k})=\sum _{i=1}^{k}\,{(-1)}^{i-1}\sum _{I\subset [k];\,card(I)=i}\,{H}_{i}({X}_{I}),$$

giving, for *k* = 3

$$\begin{array}{ccc}{I}_{3}({X}_{1};{X}_{2};{X}_{3}) & = & {H}_{1}({X}_{1})+{H}_{1}({X}_{2})+{H}_{1}({X}_{3})\\  &  & -\,{H}_{2}({X}_{1},{X}_{2})-{H}_{2}({X}_{1},{X}_{3})-{H}_{2}({X}_{2},{X}_{3})\\  &  & +\,{H}_{3}({X}_{1},{X}_{2},{X}_{3})\end{array}$$

where *k* is the number of genes analyzed as a *k*-tuple and *X*_*I*_ denotes the joint-variable corresponding to the subset *I*. I_k_ is equal to H_k_ for *k* = 1, is always non-negative for *k* < 3 and can take negative values for *k* ≥ 3^42,43^. To better understand these concepts, theoretical examples of 3 binary variables sharing maximal (positive) or minimal I_3_ (negative) are represented at different dimensions in Fig. 3b (see also Supplementary Fig. 5). While the maxima of I_3_ correspond to a fully redundant behavior, the minima of I_3_ coincide with cases where variables are pairwise independent (I_2_ = 0) but strictly tripletwise dependent. In other terms, positive I_k_ captures statistical dependences with co-variations including usual linear correlations as a subcase, zeros of I_k_ capture statistical *k*-independence, and negativity captures more complex relationships that cannot be detected in lower dimensional projections, which we named dimension-specific segregating patterns. Therefore, depending on I_k_ positivity or negativity, I_k_ analysis captures high-dimensional statistical dependences of different natures: co-variation *vs* dimension-specific segregation.

Due to computational constraints^41^, we limited our I_k_ analysis to the 21 most relevant genes (Fig. 3c), based on their level of expression and their implication in DA neuron function (Supplementary Figures 1 and 3). While both H_k_ and I_k_ were computed, we will present here only I_k_, which identifies high-dimensional statistical dependences. We first estimated I_k_ at all dimensions *k* and for every *k*-tuple of genes (for *k* ≤ *n* = 21, *n* being the total number of genes analyzed) and then computed its landscape, corresponding to the distribution of I_k_ as a function of dimension *k* (Fig. 3d–f, see Methods). I_k_ landscapes therefore represent information topological structures capturing the statistical dependences between pairs, triplets, quadruplets, etc, of genes^41^ (see Methods). In order to determine which I_k_ values could be considered as statistically significant, we performed within-cell permutations of gene expression levels (shuffling) and only considered the top or bottom 5% of the shuffled landscape as biologically relevant^41^ (Fig. 3d–f, see Methods). We also calculated the undersampling dimension *k*_*u*_ above which the estimation of H_k_ and I_k_ values should not be trusted (*k*_*u*_ = 6 and 4 for DA and nDA neurons, respectively, see Methods).

### I_k_ analysis reveals high-dimensional co-varying and segregating patterns of gene expression

The main difference between DA and nDA landscapes was the larger proportion of positive I_k_ in DA neurons and negative I_k_ in nDA neurons, respectively, above *k* = 3: for instance, the number of significant positive I_2_, I_3_ and I_4_ values is much larger in DA neurons, suggesting the presence of 2-, 3- and 4-dimensional co-varying gene modules (Fig. 3d). In contrast, the I_k_ landscapes obtained for the other 20 “non-relevant” genes were very close to *k*-independence (I_k_ = 0), demonstrating that these genes fail to provide significant information about the high-dimensional structure of gene expression in DA neurons (Fig. 3e). Consistent with the theoretical examples presented in Fig. 3b, the maximal (positive) and minimal (negative) significant I_k_ values were associated with co-varying and segregating patterns of expression, respectively (Fig. 3f). Interestingly, two of the maximal I_4_ values identified co-varying quadruplets containing DA metabolism and ion channel genes (*Drd2*/D2R, *Kcnj6*/GIRK2, *Slc6a3*/DAT, *Th*/TH; *Slc6a3*/DAT, *Kcnj6*/GIRK2, *Kcnd3*/Kv4.3, *Scn2a1*/Nav1.2; Fig. 3f). By contrast segregating patterns (negative I_k_) involved genes such as *Gad2*/GAD65, *Calb1*/CB or *Cacna1g*/Cav3.1, previously identified as heterogeneously expressed in midbrain DA subpopulations^21,44,45^ (Fig. 3f). Moreover, segregating I_4_ gene modules may also correspond to the superposition of negative and positive I_3_ (see example in Supplementary Fig. 6).

**Figure 4:**
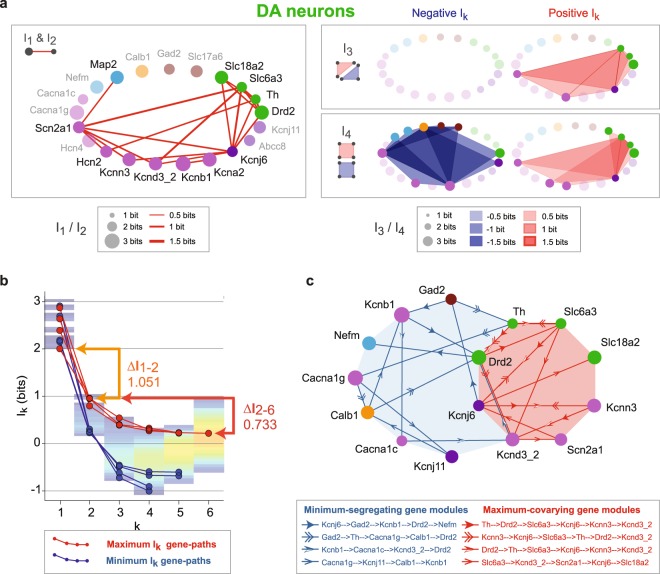
Size and stability of co-varying and segregating gene modules in DA neurons. (**a**) Scaffold representations of the most significant I_k_ values shared by pairs (I_2_, 20 examples), triplets (I_3_, 5 ex.) and quadruplets (I_4_, 5 ex.) of genes in DA neurons. Circle diameters are scaled according to entropy value (I_1_). The red shapes indicate positive I_k_ shared by genes while the blue shapes correspond to negative I_k_. Negative I_3_ triplets are not represented due to their lack of statistical significance. (**b**) Line and scatter plot illustrating the 4 maximum (red) and minimum (blue) I_k_ gene paths corresponding to stable information modules identified using conditional I_k_ computation. The total information landscape (transparent color coding) is shown in the background. The bars and arrows on the right indicate the information gain attributable to pairwise (orange) and higher-order interactions (red) for the first maximum gene-path. (**c**) Gene-scaffold representation of the identified maximum-covarying (red) and minimum-segregating (blue) I_k_ gene paths. Each path, representing a gene module, is distinguished by a specific arrowhead shape (see legend box).

We summarized the most significant positive and negative I_k_ values in DA neurons (corresponding to the most relevant gene interactions), up to *k* = 4, in a gene scaffold representation (Fig. 4a, see Supplementary Fig. 7 for the same analysis on nDA neurons). Interestingly, compared to the Pearson correlations (Fig. 2), I_2_ analysis revealed new pairs of genes, due to the fact that I_k_ also identifies non-linear dependences^46^. In addition, in DA neurons the I_2_, I_3_ and I_4_ positive I_k_ modules displayed dense overlap, meaning that pairs of co-varying genes are in fact part of co-varying triplets and quadruplets, including the DA metabolism genes and several ion channels (Fig. 4a). Therefore I_k_ analysis revealed three important aspects of gene expression profile in DA neurons. First, high-dimensional co-varying gene modules are present and seem to persist across dimensions, indicating co-expression of specific genes conserved across the whole neuronal population. Second, high-dimensional segregating gene modules are identified, corresponding to subpopulation-defining genes. Third, both types of high-dimensional gene modules coexist.

### Determining the size of regulatory modules using I_k_ analysis

Based on the observed stability of positive I_k_ modules, we wondered whether we could determine the size of the most significant positive and negative I_k_ gene modules. To do so, we calculated the changes in information content of a gene module when adding a new gene, thus defining information paths (Fig. 4b). For a given information path, the first derivative with respect to the dimension *k* is given by the conditional mutual information with a minus sign:

$$I({X}_{1};\,\mathrm{...};\,{X}_{k})\,=I({X}_{1})-\sum _{i=2}^{k}\,{X}_{i}\cdot \,H({X}_{1},\,\mathrm{...},\,{X}_{i-1})$$

$${X}_{i}\cdot \,{I}_{k-1}({X}_{1};\,\mathrm{...};\,{\hat{X}}_{i};\,\mathrm{...};\,{X}_{k})={I}_{k-1}({X}_{1};\,\mathrm{...};\,{\hat{X}}_{i};\,\mathrm{...};\,{X}_{k})-{I}_{k}({X}_{1};\,\mathrm{...};\,{X}_{k}),$$

where ^ denotes the omission of X_i_ (the conditioning variable). X_i_.I_k-1_ stays positive (negative slope) if adding a variable X_i_ (a gene) to the module increases the information while a negative X_i_.I_k-1_ (positive slope) indicates that adding a variable increases the uncertainty about the module. Therefore, reaching the equilibrium point X_i_.I_k-1_ = 0 allows to provide a measure of the size of a gene module based on the progression of the information content across dimensions^41^ (see Methods).

We restricted our search to the paths that maximized (strong positive I_k_) or that minimized mutual information (strong negative I_k_,), and we selected the 4 longest paths of each kind (Fig. 4b). For positive information paths, the first three paths identified the same 6-tuple of genes (*Th*/TH, *Drd2*/D2R, *Slc6a3*/DAT, *Kcnj6*/GIRK2, *Kcnn3*/SK3, *Kcnd3_2*/Kv4.3). In contrast, all negative paths were composed of distinct 4- or 5-tuples of genes. Interestingly, comparing the gain of information conveyed by considering pairwise interactions or higher-order interactions for the first maximal path (1.051 *vs* 0.733 bits) revealed that approximately one third of the information is gained by considering higher-than-pairwise interactions (41%, Fig. 4b). We then represented these paths in the same gene scaffold (Fig. 4c). As a confirmation of our previous results, the positive I_k_ module built using the maximal I_k_ paths contained the 4 genes involved in DA metabolism (*Th*/TH, *Drd2*/D2R, *Slc6a3*/DAT, *Slc18a2*/VMAT2) and 4 ion channels (*Scn2a1*/Nav1.2, *Kcnj6*/GIRK2, *Kcnn3*/SK3, *Kcnd3_2*/Kv4.3, Fig. 4c). Conversely, the negative I_k_ module based on minimal I_k_ paths contained a number of genes known for being heterogeneously expressed in midbrain DA neurons (*Calb1*/CB, *Cacna1g*/Cav3.1, *Kcnj11*/Kir6.2, *Gad2*/GAD65). Interestingly, this representation also revealed the intricacy of positive and negative I_k_ modules, since some of the genes are involved in both maximal and minimal paths (*Drd2*/D2R, *Kcnj6*/GIRK2, *Th*/TH and *Kcnd3_2*/Kv4.3). In other words, considering the multidimensional space demonstrates that some of the genes are involved in defining both the high-dimensional homogeneity and heterogeneity of the midbrain DA neuron population.

## Discussion

Since it was discovered that neurodegeneration in Parkinson’s disease is not homogeneous across the midbrain DA neurons of the VTA and SNc^21,47^, the heterogeneity of this neuronal population has been the subject of many studies^22,48^. For instance, it was demonstrated that the most vulnerable neurons seem to be devoid of calbindin (*Calb1*/CB). Then other differentially expressed proteins, including ion channels, have been postulated to explain, at least partially, the selective neurodegeneration of subpopulations of midbrain DA neurons^21–23,47,49^. From an electrophysiological point of view though, these neurons appear rather homogeneous and are unambiguously identified when performing *in vitro* recordings, because of their slow pacemaking, broad action potential and large hyperpolarization-induced sag^19,20^. As with any type of neuron, there is variability in the electrophysiological parameters that can be measured^5^, but this variability does not prevent discrimination from the neighboring SNr GABAergic neurons. In spite of this observation, very few studies have been dedicated to the investigation of the source of this electrophysiological homogeneity and stability.

In the present study, using sc-RTqPCR, we demonstrated that several ion channels involved in the control of spontaneous activity (*Scn2a1*/Nav1.2, *Kcnd3*/Kv4.3, *Kcnn3*/SK3) or DA response (*Kcnj6*/GIRK2) have co-varying levels of expression. Moreover, the levels of expression of these ion channels also co-vary with the levels of expression of several genes defining DA metabolism (*Th*/TH, *Slc6a3*/DAT, *Drd2*/D2R, *Slc18a2*/VMAT2). Using a newly developed mathematical analysis (I_k_ analysis), we were able to demonstrate that these co-variations are high-dimensional, involving 4 or more genes (up to 6), and co-exist with high-dimensional segregating patterns of expression of other genes (Fig. 4c). As we will discuss, these findings bring several new insights into our understanding of gene regulation and electrical phenotype genesis and stability in midbrain DA neurons.

**Figure 5:**
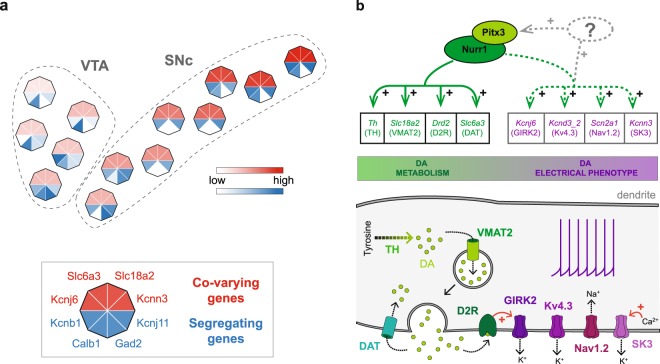
New insights into midbrain DA neuron definition. (**a**) Schematic representing the superimposition of profiles of expression of positive I_k_-sharing genes (red, co-varying) and negative I_k_-sharing genes (blue, heterogeneous) in midbrain DA neurons revealed by I_k_ analysis. While the levels of expression of positive I_k_-sharing genes vary in a homogeneous manner, the levels of expression of negative I_k_-sharing genes are extremely heterogeneous (see justification in Supplementary Fig. 8), producing a mosaic-like pattern. (**b**) Top, schematic representation of the documented transcriptional co-regulation of DA metabolism genes (green) by the transcription factors Nurr1 and Pitx3, together with the potential co-regulation of the genes involved in electrical phenotype (purple) identified in the present study. The functional implication of the corresponding proteins in DA signaling pathway (green shading) and DA neuron electrical properties (purple shading) are represented in the schematic below. The potential coupling of these two groups of genes may reflect a basic regulatory functional module common to all midbrain DA neurons.

One important result of the present study is that, in some respects, midbrain DA neurons appear highly homogeneous. The neurons collected in the present study came from acute dissociation of midbrains of TH-GFP mice, and therefore originate from both the VTA and the SNc. The I_k_ analysis identified high-dimensional co-variations of gene expression levels (positive I_k_ modules, Fig. 4) from the cell-to-cell variability of expression observed in the 111 neurons identified as DA. This means that, although each of the genes belonging to these modules has a variable level of expression, their quantitative relationship is conserved across all DA neurons (see red inset in Fig. 3f). In other words, since these co-variations appear more or less linear, this means that the ratio of all the positive I_k_ module genes (*Scn2a1*/Nav1.2, *Kcnd3_2*/Kv4.3, *Kcnn3*/SK3, *Kcnj6*/GIRK2, *Th*/TH, *Slc6a3*/DAT, *Drd2*/D2R, *Slc18a2*/VMAT2, Fig. 4c) is kept roughly constant in all midbrain DA neurons, whether originating from the VTA or the SNc. Interestingly, some of these genes have been previously demonstrated to vary across the VTA and SNc, and have been suspected to delineate DA neuron subpopulations (Supplementary Fig. 8). For instance, Wolfart and colleagues have suggested that SK3 (*Kcnn3*) expression level is higher in lateral SNc than medial SNc^18^, and *Drd2*/D2R has been proposed to be more expressed in SNc than in VTA^48^. This is compatible with our results, as we show that the co-varying genes display substantial cell-to-cell variations in their expression levels. Our results refine these observations by stating that the variability in expression of these genes is continuous^50^. Interestingly, *Drd2*/D2R was found to be part of both positive and negative I_k_ modules (Fig. 4c), therefore participating in both homogeneous and heterogeneous patterns of expression. Altogether, this suggests that the co-varying genes (positive I_k_ module) may display a medio-lateral gradient, their expression levels being lowest in the VTA and highest in the lateral SNc (Fig. 5a, Supplementary Fig. 8), reminiscent of recent observations made in the hippocampus^51^.

The I_k_ analysis also identified several genes involved in dimension-specific segregating patterns (Fig. 4c). In other words, subpopulations of neurons are revealed only by the simultaneous consideration of the expression levels of more than 3 genes (see blue inset in Fig. 3f). This can be explained in the following way: the genes involved in these segregating patterns display multimodal expression patterns (such as a low and a high peak of expression, Supplementary Fig. 3), and these slight discontinuities in expression “add up” to create clear clustered patterns of expression in 3 or 4 dimensions (Fig. 3f and Supplementary Fig. 6) corresponding to neuron subpopulations. For instance, the two main clusters in Fig. 3f (left plot) delineate subpopulations of DA neurons expressing low Calb1-high Kcnb1-low Gad2 or high Calb1-high Kcnb1-high Gad2. Interestingly, many of these genes have already been proposed to define midbrain DA neuron subpopulations, for instance *Calb1*/CB, *Cacna1g*/Cav3.1 or *Gad2*/GAD65 (Supplementary Fig. 8)^21,44,45^. Our results confirm these observations and extend them by demonstrating that considering one or two genes might not be enough to distinguish these subpopulations, and that high-dimensional statistical tools are necessary to unravel this heterogeneity. Moreover, I_k_ analysis identifies new “segregating” genes, such as the voltage-gated potassium channel subunit *Kcnb1*/Kv2.1. Determining the precise number of subpopulations of DA neurons corresponding to these clustering patterns would require to compute I_k_ on the transpose matrix (using cells as variables and genes as observations), which unfortunately is currently computationally untractable^41^.

We therefore suggest that midbrain DA neurons simultaneously host two types of variability in their gene expression levels: *i)* some of the genes display cell-to-cell “continuous” variability in expression and might be mainly involved in high-dimensional co-varying expression patterns, revealing conserved regulatory rules among all midbrain DA neurons and *ii)* other genes display cell-to-cell “discontinuous” variability in expression and might be mainly involved in dimension-specific segregating patterns of expression, identifying subpopulations of midbrain DA neurons. As a result, midbrain DA neurons appear simultaneously homogeneous and heterogeneous, depending on the genes that are analyzed and the high-dimensional statistical dependences that they share (Fig. 5a, see also example in Supplementary Fig. 6). Moreover, since several genes of the co-varying module are involved in DA metabolism, the homogeneous co-variation of the positive I_k_ genes may correspond to a medio-lateral gradient of increasing DA “nature”.

The most significant result of the present study is that neurotransmitter identity and electrophysiological phenotype are genetically coupled in midbrain DA neurons. The high-dimensional co-varying gene module identified with the I_k_ analysis links DA metabolism and ion channels: *i)* four DA genes (*Th*/TH, *Slc18a2*/VMAT2, *Slc6a3*/DAT, *Drd2*/D2R) involved in DA synthesis, vesicular uptake, plasma membrane uptake, and synaptic signaling, respectively *ii)* three voltage- and calcium-gated ion channels (*Scn2a1*/Nav1.2, *Kcnd3*/Kv4.3, *Kcnn3*/SK3) involved in the genesis and control of spontaneous activity and *iii)* one G protein-coupled ion channel (*Kcnj6*/GIRK2) known to be an effector of the auto-receptor *Drd2*/D2R in dendro-dendritic release of DA^21,23,52^. This finding is interesting in several respects and may have several interpretations.

At this point, it is important to remember what the I_k_ analysis tells us about gene regulatory principles. While most gene regulatory networks have been inferred using pairwise analysis of gene expression levels^36^, complex regulatory mechanisms, such as the cooperative actions of two (or more) transcription factors, can only be accurately captured by a “truly” high-dimensional analysis^37,39^. In fact, theoretically, the size of a genetic regulatory module can only be determined by an analysis that reaches the dimensionality of the module (the number of genes comprising it). I_k_ analysis was performed up to the 21^st^ dimension, and revealed several overlapping 6-gene modules comprising a total of 8 distinct genes (Fig. 4b,c). It also revealed that a substantial part (up to 41%) of the mutual information shared by these genes appears above dimension 2 (see Fig. 4b), thus demonstrating that the 3-way, 4-way (and above) statistical dependences that pairwise analysis would not detect, significantly shape their pattern of expression. This suggests that the positive I_k_ module might be the product of complex regulatory mechanisms commonly targeting the majority of the 8 genes composing it.

Two main hypotheses can explain this. First, previous studies have demonstrated that the expression levels of *Th*/TH, *Slc6a3*/DAT, *Slc18a2*/VMAT2 and *Drd2*/D2R are under the control of the same pair of transcription factors *Nurr1* and *Pitx3*^53^. Our results moreover suggest that these four genes may be part of a larger functional module (≥6 genes) also comprising genes defining the electrical properties of DA neurons (such as *Kcnj6*/GIRK2, *Kcnd3_2*/Kv4.3, *Kcnn3*/SK3, *Scn2a1*/Nav1.2) (Fig. 5b). Alternatively, but not exclusively, this coupling might reflect the documented activity-dependent regulation of DA-specific genes such as *Th*/TH, which has been shown to be sensitive to blockade of sodium (including *Scn2a1*/Nav1.2) and potassium channel activity^54^ (including *Kcnn3*/SK3). Consistent with this idea, modeling studies suggested that co-varying profiles of expression of ion channels^9,10^ could be the consequence of homeostatic plasticity of gene expression^15^. Interestingly, in these studies, simple homeostatic rules linking calcium variations to ion channel expression systematically produced linear co-variations of ion channel levels, independent of the sign or rate of regulation of each gene. Moreover, these co-variations of ion channel expression were associated with a constancy of the electrical phenotype of the model neurons. While we have not tested (experimentally or computationally) this hypothesis, we postulate that the co-variations in ion channel expression (*Scn2a1*/Nav1.2, *Kcnd3*/Kv4.3, *Kcnn3*/SK3, *Kcnj6*/GIRK2) observed in our experiments may play a homeostatic role and ensure that a correct electrophysiological phenotype is achieved and maintained in midbrain DA neurons. Moreover, the strong coupling we find between these ion channels and DA metabolism and signaling genes suggests that electrical phenotype and neurotransmitter identity are “set” together in this neuronal population and are under the dependence of the same regulatory processes, activity-dependent or -independent. Interestingly, while theory predicts that negative co-variations of ion channels may arise from homeostatic rules^15^, especially for functionally overlapping ion channels, we only observed positive co-variations. While this may seem surprising, all studies performed so far at the single-cell level identified only positive co-variations of ion channel expression, independent of the system investigated^9–11^. Nevertheless, we cannot rule out that negative co-variations may exist between ion channels that have yet to be analyzed.

Another important aspect is that most (if not all) of the co-varying module genes seem to correspond to dendritic proteins (Fig. 5b): *Kcnd3*/Kv4.3 and *Kcnn3*/SK3 have been shown to be located in dendrites^30,55^, sodium channels are present in dendrites^56^ (including potentially *Scn2a1*/Nav1.2), and *Kcnj6*/GIRK2 is the effector of *Drd2*/D2R exclusively at dendro-dendritic DA synapses^57^. As shown in Fig. 2c and Fig. 4a, *Map2*/MAP2 and *Scn2a1*/Nav1.2 expression levels significantly correlate or co-vary, and most of the genes cited above also share significant correlations with *Map2*/MAP2 (*Kcnd3*/Kv4.3, *Kcnn3*/SK3, *Kcnj6*/GIRK2 and *Drd2*/D2R; 0.576 < r < 0.629, n = 111, p < 0.05, data not shown). This suggests that the gene expression levels of these dendritic proteins might scale as a function of dendritic length.

In the present study, we only analyzed the levels of expression of 41 genes, and missed many genes that are central to DA neuron function. In particular, we did not cover the entire pool of ion channels expressed by DA neurons. Moreover, stability of electrophysiological phenotype can only be understood if neuronal output is examined simultaneously with ion channel expression. Also, although ion current amplitude has been shown to scale with mRNA expression level in several neuronal types for several ion channels^7,8,58,59^ (including A-type potassium current and *Kcnd3*/Kv4.3 in SNc DA neurons), many regulatory steps downstream gene expression can modulate protein function. Defining the influence of variations in ion channel expression on electrical phenotype will necessitate an experimental consideration of these post-mRNA regulatory processes^60^. In spite of these caveats, we demonstrated for the first time that electrophysiological phenotype (ion channel expression) is tightly genetically coupled with neurotransmitter identity (DA metabolism genes), and likely to be controlled by the same regulatory processes. Combining patch-clamp recordings with single-cell transcriptomics as has been done in other structures^61^ will undoubtedly be a necessary task if we want to get a clear understanding of the molecular principles underlying the physiological stability (and pathophysiological instability) of activity in midbrain DA neurons.

## Material and Methods

### Acute midbrain slices preparation

Acute slices were prepared from P14–P23 TH-GFP mice (transgenic mice expressing GFP under the control of the tyrosine hydroxylase promoter)^62^ of either sex. All experiments were performed according to the European Directive 2010/63/EU of the European Parliament and to the French national law implementing this directive (orders *AGRG1231951D; AGRG12400332A; AGRG1238753A; AGRG1238729A; AGRG1238767A; AGRG1238724A*). The experiments described are based on the use of organs and tissues collected from dead animals after euthanasia, and therefore do not require a specific authorization as stated by French law. Mice were anesthetized with isoflurane (Piramidal Healthcare Uk) and decapitated. The brain was immersed briefly in oxygenated ice-cold low calcium artificial cerebrospinal fluid (aCSF) containing the following (in mM): 125 NaCl, 25 NaHCO_3_, 2.5 KCl, 1.25 NaH_2_PO_4_, 0.5 CaCl_2_, 4 MgCl_2_, 25 glucose, pH 7.4, oxygenated with 95% O_2_/5% CO_2_ gas. The cortices were removed and then coronal midbrain slices (250 μm) were cut on a vibratome (Leica VT 1200 S) in oxygenated ice-cold low calcium aCSF. Following 30–45 min incubation in 32 °C oxygenated low calcium aCSF, the slices were incubated for at least 30 min in oxygenated aCSF (125 NaCl, 25 NaHCO_3_, 2.5 KCl, 1.25 NaH_2_PO_4_, 2 CaCl_2_, 2 MgCl_2_ and 25 glucose, pH 7.4, oxygenated with 95% O_2_/5% CO_2_ gas) at room temperature prior to electrophysiological recordings. Picrotoxin (100 μM, Sigma Aldrich, St. Louis, MO) and kynurenate (2 mM, Sigma Aldrich) were bath-applied via continuous perfusion in aCSF to block inhibitory and excitatory synaptic activity, respectively.

### Cell dissociation and collection

Midbrain DA neurons were acutely dissociated following a modified version of the methods described in references^63^ and^24^. Regions containing the SNc, part of the VTA and SNr were excised from each coronal midbrain slice. The tissue was submitted to papain digestion (2.5 mg/ml and 5mM L-cysteine) for 15–20 min in oxygenated low calcium HEPES aCSF (containing 10 mM HEPES, pH adjusted to 7.4 with NaOH) at 35–37 °C and subsequently rinsed in low-calcium HEPES aCSF supplemented with trypsin inhibitor and bovine serum albumin (1 mg/ml). Single cells were isolated by gentle trituration with fire-polished Pasteur pipettes and plated on poly-L-Lysine-coated coverslips. Dissociated cells were maintained in culture in low calcium HEPES-aCSF at 37° in 5% CO_2_ for at least 45 minutes. Coverslips were then placed in a cell chamber of a fluorescence microscope and continuously perfused with HEPES-aCSF. Cells were collected by aspiration into borosilicate glass pipettes mounted on a micromanipulator under visual control. Cell dissociation and collection were performed using RNA-protective technique and all solutions were prepared with RNase-free reagents when possible and filtered before use.

### Electrophysiology recordings, data acquisition and analysis

All recordings were performed as already described previously^6^. Picrotoxin and kynurenate were present for all recordings to prevent contamination of the intrinsic activity by spontaneous glutamatergic and GABAergic synaptic activity. Statistical analysis (performed according to data distribution) included: unpaired *t* test, Mann Whitney, paired *t* test with a p value < 0.05 being considered statistically significant. Statistics were performed utilizing SigmaPlot 10.0 (Jandel Scientific, UK) and Prism 6 (GraphPad Software, Inc., La Jolla, CA).

### qPCR assays, specific retro-transcription and targeted amplification (RT-STA)

Pre-designed TaqMan assays (TaqMan® Gene Expression Assays, Thermo Fisher Scientific) used in this study are listed in Supplementary Table 1. Assays were systematically selected to target the coding region and to cover all known splice variants. In the case of *Kcnd3* and *Kcnj6* genes, two different assays were used to detect all known splice variants. Excluding *Fos* (754 bp intron) and *Bdnf*, *Kcna2* and *Kcnj11* (both primers and probe within a single exon), assays spanning a large intron (>1000 bp) were chosen to avoid genomic DNA amplification. *Gad1* primers and probe were designed according to Applied Biosystems criteria and MIQE recommendations^64^ TaqMan® assays were pooled (0.2x final concentration) and the preamplification step was validated using log serial dilutions of mouse brain total RNA (MBTR). The following thermal profile was applied: 50 °C for 15 min, 95 °C for 2 min and 22 cycles of amplification^65^ (95 °C for 15 s and 60 °C for 4 min) following Fluidigm recommendations. For each assay, efficiency was estimated from the slope of the standard curve using the formula E = (10^(−1/slope)^ − 1) ×100. All assay efficiencies (89.4 ≤ E ≤ 100.4%) are listed in Supplementary Table 1.

### Single-cell RTqPCR, data processing and analysis

Individual GFP and non-GFP neurons were harvested directly into 5 µl of 2x Reaction buffer (CellsDirect™ One-Step qRTPCR, Lifetech) and kept at −80 °C until further processing. A reverse transcription followed by a specific targeted pre-amplification (RT-STA) was performed in the same tube (2.5 µl 0.2x assay pool; 0.5 µl SuperScript III) applying the same thermal profile described above. The pre-amplified products were treated with ExoSAPI (Affimetrix) and diluted 5-fold prior to analysis by qPCR using 96.96 Dynamic Arrays on a BioMark System (BioMark™ HD Fluidigm). Data were analyzed using Fluidigm Real-Time PCR Analysis software (Linear Baseline Correction Method and User detector Ct, Threshold Method). Two genes, *Kcnj6_c* and *Chat* were undetectable in all analyzed cells. Cells that had a Ct for *Hprt* above 21 were excluded from further analysis. After interplate calibration, all Ct values were converted into relative expression levels using the equation Log_2_Ex = Ct_LOD_ − Ct_(Assay)_^66^. LOD (limit of detection) was set to Ct = 25 by calculating the theoretical Ct value for 1 single molecule in the Biomark system from two custom-designed oligonucleotides: *Slc18a2* and *Penk*. All data pre-processing was performed in Microsoft Excel (Microsoft, Redmond, USA). Heatmap and correlation maps (Pearson correlation coefficient values excluding zero values, p value ˂0.05, n ˃ 5) were generated in the R environment (R Core Team 2016) using gplots, heatmap3, Hmics and corrplot packages. Gene expression scatter plots and frequency distribution plots were created in SigmaPlot 10.0 (Jandel Scientific) and Prism 6 (GraphPad Software, Inc, La Jolla, CA). Figures were prepared using Adobe Illustrator CS6.

### Topological information data analysis

The present analysis is based on the information cohomology framework developed by Baudot and Bennequin^40^ that characterizes uniquely entropy and multivariate mutual information (I_k_) and develops the topological computational framework for the data analysis^41^.

#### Information functions

The information functions provide the general random variable lattice of joint-variables. The application of this framework to data analysis is developed in the subcase of simplicial information homology, (see^41^ for more detail), whose exploration follows binomial combinatorics with a complexity in *O*(2^n^). It allows an exhaustive estimation of the information structure of the gene expression, that is the joint-entropy H_k_ and the mutual information I_k_, on all degrees *k* and for every *k*-tuple of variables (gene expression levels), defined respectively by the following equations:

$${H}_{k}\,=\,H({X}_{1},\,\mathrm{...},{X}_{k};\,{P}_{{X}_{1},\mathrm{...},{X}_{k}})\,=\,k\sum _{{x}_{1},\,\mathrm{...},{x}_{k}\,\in \,[{N}_{1}\times \,\mathrm{...}\times {N}_{k}]}^{{N}_{1}\times \,\mathrm{...}\times {N}_{k}}p({x}_{1}\,\mathrm{.....}{x}_{k})\,\mathrm{ln}\,p({x}_{1}\,\mathrm{.....}{x}_{k})$$

$${I}_{k}({X}_{1};\,\mathrm{...};{X}_{k})\,=\,\sum _{i=1}^{k}{(-1)}^{i-1}\sum _{I\subset [k];card(I)=i}{H}_{i}({X}_{I})$$

for a probability joint-distribution P_X1,…,Xk_ and joint-random variables (X_1_, …, X_k_) with alphabet [N_1_…N_k_] and k = −1/ln2, where *n* variables are mutually independent if and only if ∀ *k* ≤ *n*, I_k_ = 0. Due to the combinatorial complexity, in the current study H_k_ and I_k_ values were computed for *n* = 21 (for *n* = 21, the total number of information elements to estimate is 2 097 152). The landscapes are representations of the information structures where each element of the lattice is represented as a function of its corresponding value of entropy or mutual information, and quantify the variability-randomness and statistical dependences at all degrees *k*, respectively, from 1 to *n* (see ref.^41^ for more detail). The distributions of I_k_ for every degree *k* (corresponding to *k*-tuples of gene expression levels) were represented as I_k_ landscapes in Fig. 3.

#### Probability estimation

The probability estimation procedure is explained in detail in^41^ for the simple case of two random variables (the expression levels of two genes). For each variable *X*_*j*_, we consider the space in the intervals [min *x*_*j*_, max *x*_*j*_] and divide it into *N*_*j*_ boxes, *N* being the graining of the data. The empirical joint probability is estimated by box counting after a graining of the data space into *N*_*1*_*…N*_*k*_ boxes (for *k*-tuple probability estimation). In the current study, a graining of *N*_*1*_ = … = *N*_*k*_ = 9 was chosen as it provided a correct description of the distribution of the expressions levels.

#### Information paths

An information path IP_k_ of degree *k* on I_k_ landscape is defined as a sequence of elements of the lattice that begins at the least element of the lattice (the identity-constant “0”), travels along edges from element to element of increasing degree of the lattice and ends at the greatest element of the lattice of degree *k*. The first derivative of an IP_k_ path is minus the conditional mutual information. The (“non-Shannonian”) information inequalities^42^, e.g. the negativity of conditional mutual information that quantifies the instability of the mutual information along the path, are then equivalent to the existence of local minima on such paths^41^. The critical dimension of an IP_k_ path is the degree of its first minimum. A positive information path is an information path from 0 to a given I_k_ corresponding to a given *k*-tuple of variables such that I_k_ < I_k-1_ < … < I_1_. We call the interacting components functions I_k_, *k* > 1, a free information energy. A maximal positive information path is a positive information path of maximal length: it ends at minima of the free information energy function. In the current study, the length of maximal positive information paths was considered to indicate the size of a stable information gene module. The set of all these paths defines uniquely the minimum free energy complex^41^. The set of all paths of degree *k* is intractable computationally (complexity in *O*(*k*!)). In order to bypass this issue, we used a fast local algorithm that selects at each element of degree *k* of an IP path the positive information path with maximal or minimal I_k+1_ value or stops whenever X_k_.I_k+1_ ≤ 0 and ranks those paths by their length.

#### Robustness of the method: undersampling dimension and k-independence test

Probability estimation in high dimension can be severely biased by the limited size of the sample, the number of empirical points *m*. In order to circumvent these biases we propose two statistical tests to obtain significant values in the dimension and in the information values (the abscissa and ordinates of the landscapes). To estimate the degree after which the sample size *m* becomes limiting and biases our estimations, the undersampling regime was quantified by the dimension *k*_u_ for which the probability p_u_ of having the H_k_ at the biased value of H_k_ = log_m_ is above 5 percent (*p*_*u*_ = 0.05). This basic estimation gives here *k*_*u*_ = 6 for DA neurons and *k*_*u*_ = 4 for nDA neurons, and I_k_ values beyond these degrees should be interpreted with caution.

Concerning the statistical significance of the results, we provide an extension of 2-independence test^67^ to an arbitrary *k* dimension, with the null hypothesis being the *k*-independence I_k_ = 0. We designed a shuffling procedure of the *n* variables that consists in randomly permuting the measured values (co-ordinates) of each variable one by one in the matrix *D*, leaving marginal probabilities invariant while statistical dependences between the variables should be destroyed (see discussion in ref.^41^). Our global test consists in computing 17 different shuffles of the 21 genes, giving “null” distribution of biased I_k_ values. We determine the statistical significance thresholds as information values for which the integral of the null distribution reaches the significance level *p* = 0.05. Since for *k* ≥ 3 I_k_ can be negative, the test becomes symmetric on the distribution, and for *k* ≥ 3 we chose a significance level of *p* = 0.1 in order to stay consistent with the 2-independence test. If the observed value of I_k_ is above or below these thresholds values, we reject the null hypothesis and consider the tuple as significantly *k*-dependent. The robustness of the method to the sample size (*m*) and graining value (*N*) is investigated in^41^.

#### Computation and algorithm

The Information Topology open source program, written in Python, is available on Github depository. It allows to compute the information landscapes, paths, and minimum free energy complex, which encode and represent directly all the basic equalities, inequalities, and functions of information theory^41^, and all the structures of the statistical dependences within a given set of empirical measures (up to the approximations, computational tractability and finite size biases, see previous sections). It can be run on a regular personal computer up to *k* = *n* = 21 random-variables in a reasonable time (3 hours), and provides new tools for pattern detection, dimensionality reduction, ranking and clustering based on a unified homological and informational theory.

## Electronic supplementary material

Supplementary material
