# Supplementary material for: Neurotransmitter identity and electrophysiological phenotype are genetically coupled in midbrain dopaminergic neurons
Source: Sci Rep. 2018 Sep 11;8:13637. doi: 10.1038/s41598-018-31765-z (PMC6134142; doi:10.1038/s41598-018-31765-z)
Supplement: Supplementary file 1 — Supplementary material [file 41598_2018_31765_MOESM1_ESM.pdf]

Supplementary information

Neurotransmitter identity and electrophysiological phenotype are genetically coupled in midbrain dopaminergic neurons

Mónica TAPIA[1,§], Pierre BAUDOT[1,§], Christine FORMISANO-TRÉZINY[1], Martial A. DUFOUR[1], Simone TEMPORAL[1], Manon LASSERRE[1], Béatrice MARQUÈZE-POUEY[1], Jean GABERT[1,2], Kazuto KOBAYASHI[3] and Jean-Marc GOAILLARD[1*]

[1] Unité de Neurobiologie des Canaux Ioniques et de la Synapse, INSERM UMR 1072, Aix Marseille Université, 13015 Marseille, FRANCE

[2] Département de Biochimie et Biologie Moléculaire, Hôpital Nord, Marseille, FRANCE

[3] Department of Molecular Genetics, Institute of Biomedical Sciences, Fukushima Medical University, Fukushima, 960-1295, JAPAN

[*] Corresponding author

§ These authors contributed equally to this work

Corresponding author: Jean-Marc GOAILLARD

UMR_S 1072, INSERM, Aix Marseille Université, Faculté de Médecine Secteur Nord, Marseille, FRANCE.

Email: jean-marc.goaillard@univ-amu.fr

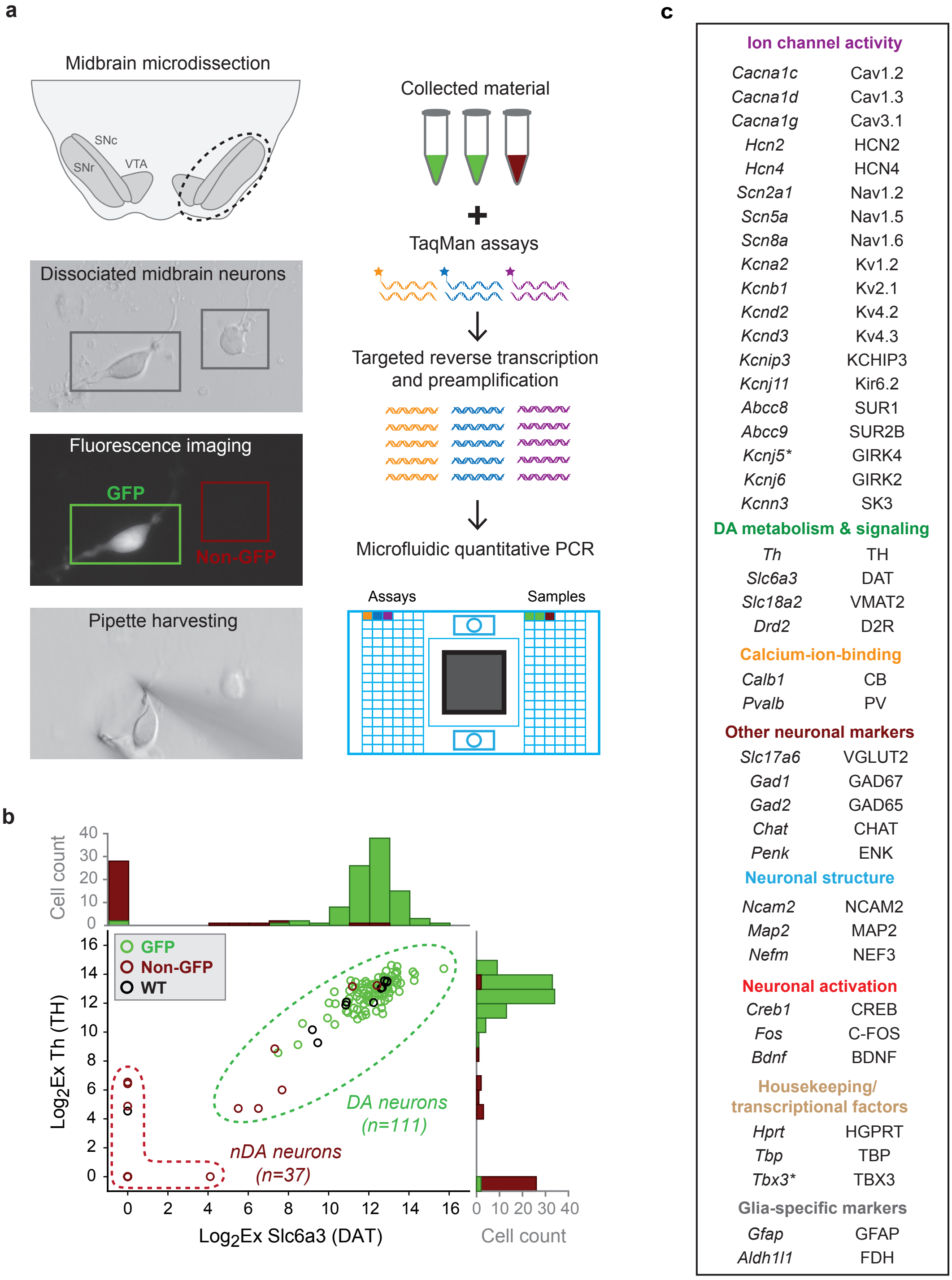

Supplementary Fig. 1. Experimental workflow and classification of DA and nDA neurons. **a**, schematic showing the workflow for the single-cell gene profiling. Left, neurons were manually collected after acute dissociation from microdissected midbrain slices containing the substantia nigra pars compacta and reticulata (SNc, SNr) and part of the ventral tegmental area (VTA) obtained from TH-GFP mice. Right, specific targeted reverse transcription and amplification was then performed in the same tube, and microfluidic qPCR was run by using 96.96 Dynamic arrays on a Biomark^{TM} HD System. **b**, DA and nDA neurons were classified based on the expression levels of *Th* (TH) and *Slc6a3* (DAT), nDA neurons corresponding to neurons expressing 0 *Th* and/or 0 *Slc6a3*. **c**, list of the genes selected for the single-cell study with the gene names (left) and corresponding protein names (right). Genes are classified based on the functional class of the corresponding proteins (colored text). Genes mainly related to cardiac cell phenotype (indicated by an asterisk), glia-specific markers and housekeeping/transcriptional factors were excluded from the subsequent Pearson analysis (**Fig. 2**).

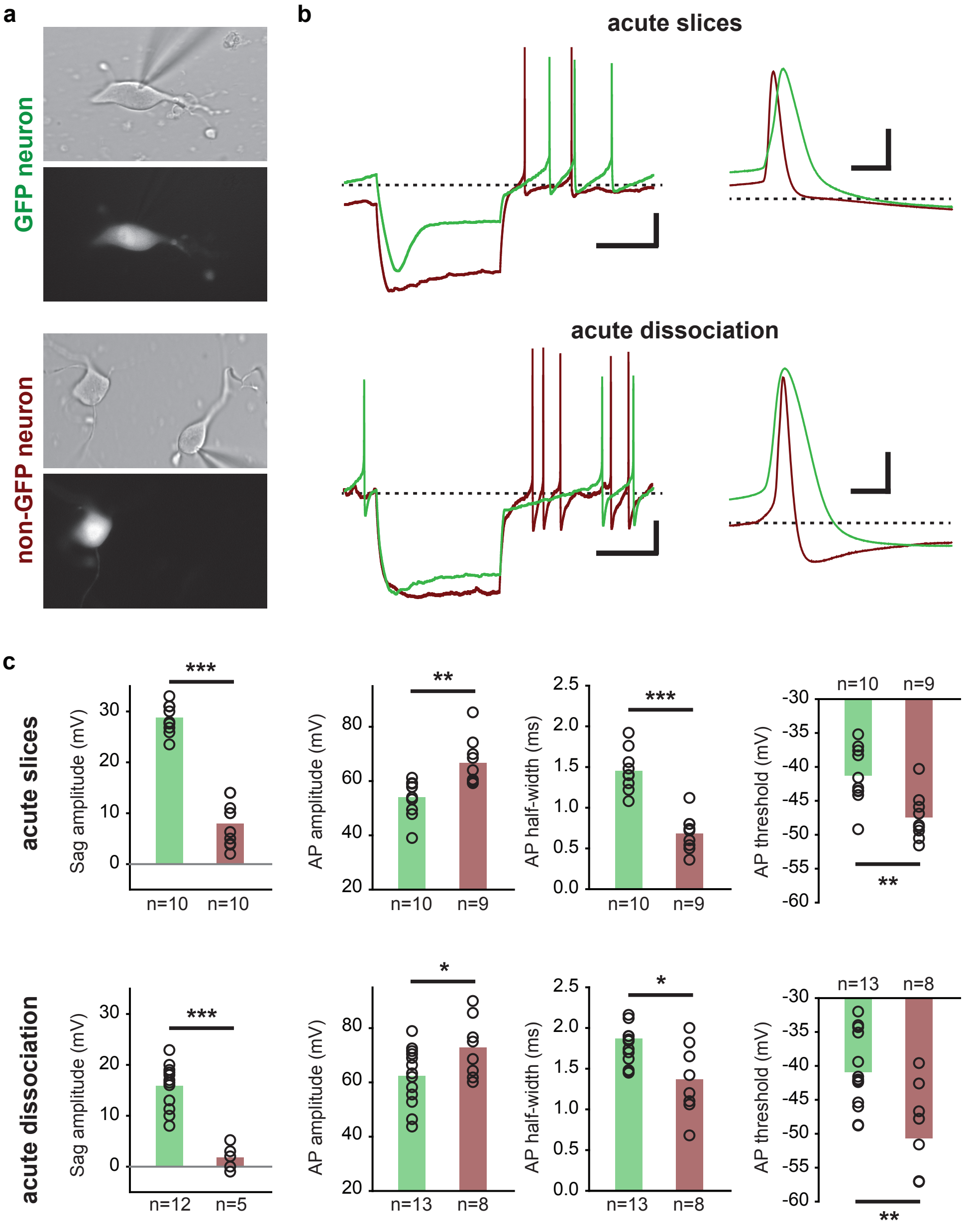

Supplementary Fig. 2. Electrophysiological properties of GFP and non-GFP midbrain neurons in acute dissociation and in acute slices. a, bright field and fluorescence images corresponding to recorded GFP (top pictures) and non-GFP neurons (bottom pictures). **b**, representative recordings obtained from GFP (green traces) and non-GFP midbrain neurons (dark red traces) in acute slices (top traces) and after acute dissociation (bottom traces). Please note the differences in sag amplitude and rebound kinetics (left traces) and the significantly broader action potential (right traces) in GFP neurons. **c**, bar and scatter plots summarizing the electrophysiological differences observed between GFP and non-GFP neurons in acute slices (top row) or after acute dissociation (bottom row). As expected for midbrain DA neurons, GFP neurons were characterized by significantly larger sag amplitude (left plot), smaller and broader action potential (middle plots), and more depolarized action potential threshold (right plot). Scale bars in **b**: 20mV, 500ms for left traces; 20mV, 2ms for right traces. Dotted lines indicate -60mV. Asterisks in **c** indicate statistical significance (Mann-Whitney). * p<0.05, ** p<0.01, *** p<0.001.

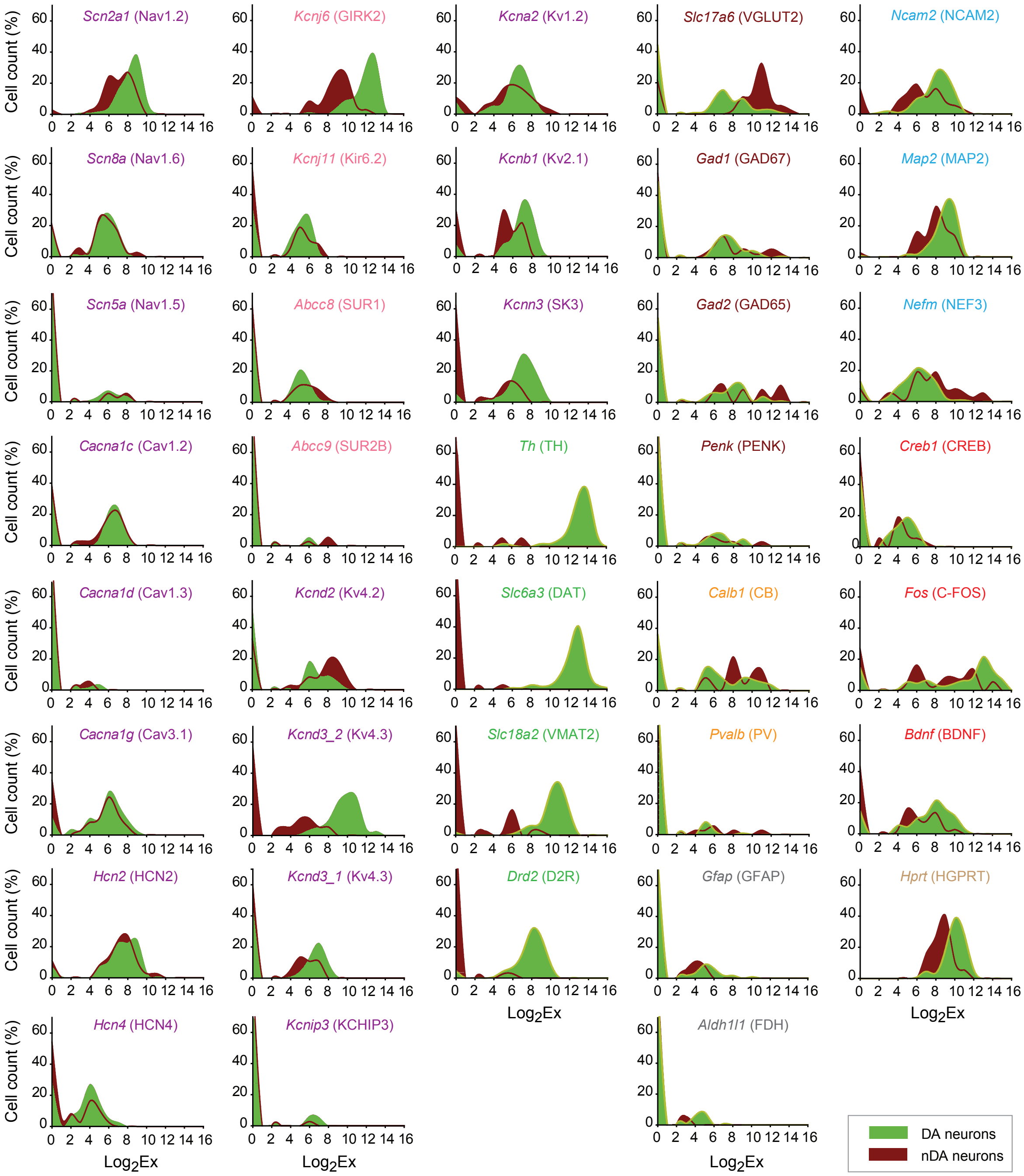

Supplementary Fig. 3. Gene expression profiles in DA and nDA neurons. Graphs showing the frequency distribution of gene expression levels in DA (green) and nDA (dark red) neurons. The three genes with the lowest expression (*Tbp*, *Kcnj5* and *Tbx3*) are not represented. The Y-axes correspond to normalized cell count (%) and the X-axes to relative gene expression levels in logarithmic (Log_2Ex) scale.

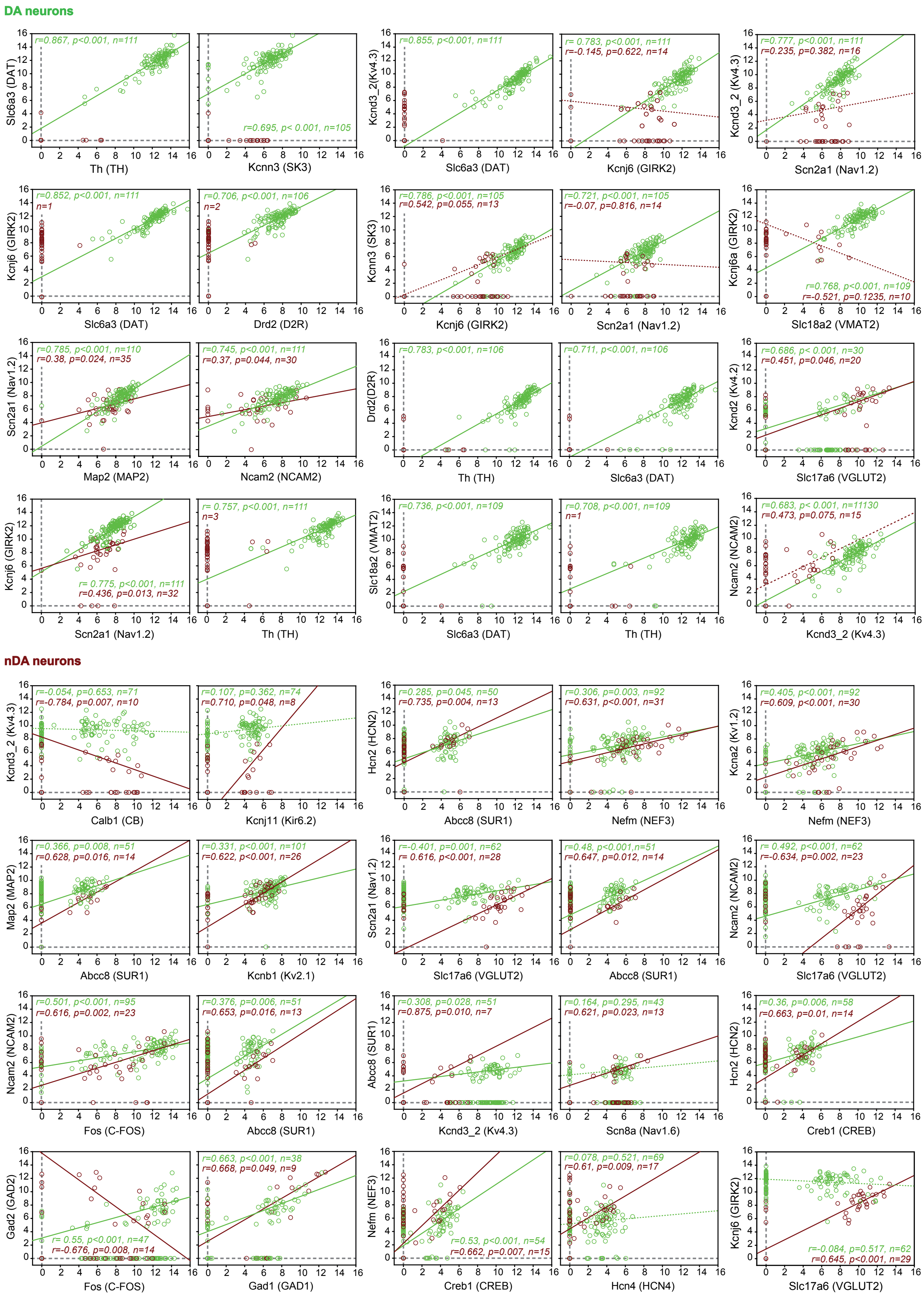

Supplementary Fig. 4. Scatter plots of the 20 most significant linear correlations in expression levels in DA and nDA neurons. Axes indicate relative gene expression level in logarithmic (Log_2Ex) scale. Green and dark red dots correspond to DA and nDA neurons, respectively. Plain and dotted lines indicate significant and non-significant Pearson correlations, respectively. *r*, *p*, and *n* values are displayed for each correlation test. Protein names corresponding to each gene are given in parentheses.

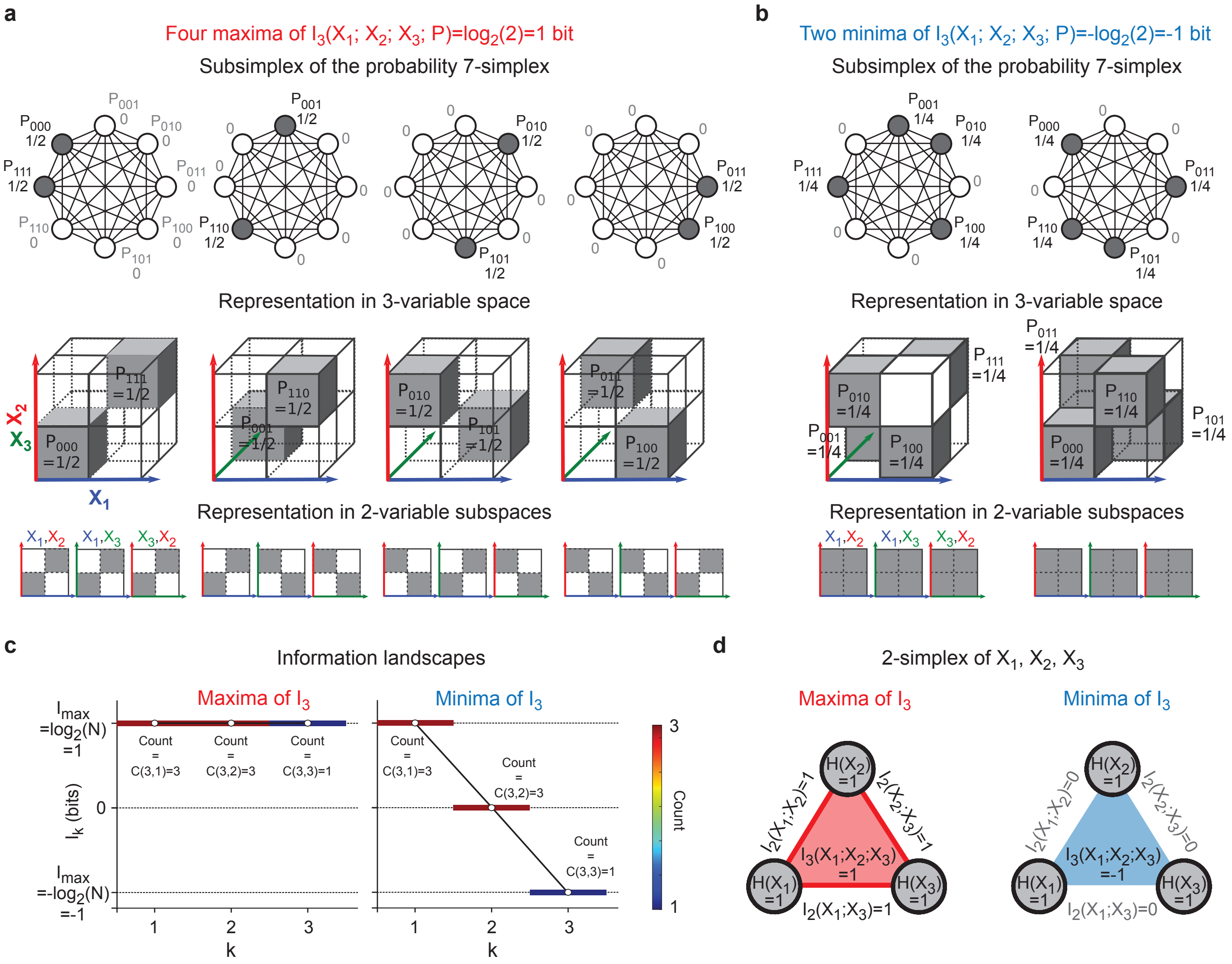

Supplementary Fig. 5. **Examples of mutual information maxima (positive) and minima (negative).** The simplest case of maxima and minima of I_3 for 3 binary random variables X_1,X_2,X_3 is represented. The corresponding probability simplex is 7-dimensional, and is depicted at the top of panels **a** and **b**. The 8 vertices are labeled by the atomic probabilities noted $P_{000},P_{001},...,P_{111}$. **a**, there are 4 maxima of I_3, $I(X_1;X_2;X_3)$= 1 bit, corresponding to the 4 subsimplex of probabilities where all atomic probabilities are 0 except for the pairs $\{P_{000}=1/2,P_{111}=1/2\}$, $\{P_{110}=1/2,P_{001}=1/2\}$, $\{P_{101}=1/2,P_{010}=1/2\}$, $\{P_{011}=1/2,P_{100}=1/2\}$. The corresponding configurations in the 3-variable space correspond to the diagonals of the cube, such that the 3 projections on the 2-variable subspaces correspond to the diagonals for which I_2 is maximal, *i.e.* $I(X_1;X_2)=I(X_1;X_3)=I(X_2;X_3)$= 1 bit, and that the projections on single-variable subspaces are maximally uncertain ($H(X_1)=I(X_1)=I(X_2)=I(X_3)$= 1 bit). **b**, the same representations as in panel **a** are shown for the two minima of I_3, $I(X_1;X_2;X_3)$= -1 bit, corresponding to the configurations where all atomic probabilities are 0 except for the 4-tuples $\{P_{001}=1/4,P_{010}=1/4,P_{100}=1/4,P_{111}=1/4\}$, $\{P_{000}=1/4,P_{011}=1/4,P_{101}=1/4,P_{110}=1/4\}$. The corresponding configurations in the 3-variable space consist of alternated volumes, such that the projections on the 2-variable subspaces are maximally uncertain, *i.e.* $I(X_1;X_2)=I(X_1;X_3)=I(X_2;X_3)$= 0 bit, and that the projections on marginal single-variable subspaces are also maximally uncertain (or informative; $H(X_1)=I(X_1)=I(X_2)=I(X_3)$= 1 bit). **c**, information landscapes associated with the maxima and minima presented in **a** and **b**. Count (n,k) gives the number of combinations for each degree k. **d**, equivalent simplicial representation with the k-faces labeled with their corresponding I_k values.

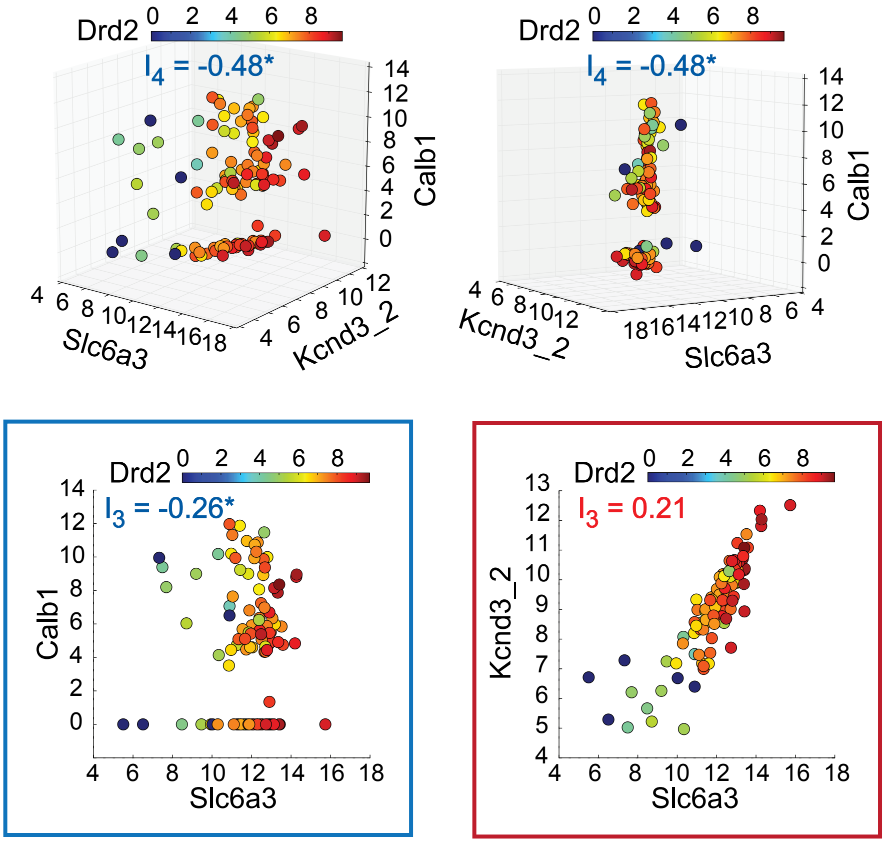

Supplementary Fig. 6. Superposition of negative and positive I_k in high dimensions. 4D- (up) and 3D-scatter plots (botton) for a quadruplet of genes sharing positive and negative I_3.The 3D-plots (up) of subsets of the original quadruplet illustrate how the gene expression profiles of DA neurons are made of the superposition of negative and positive I_k. Unsurprisingly, the mixing of positive and negative I_3 leads to non-significant I_4. Asterisks indicate non-significant I_k values.

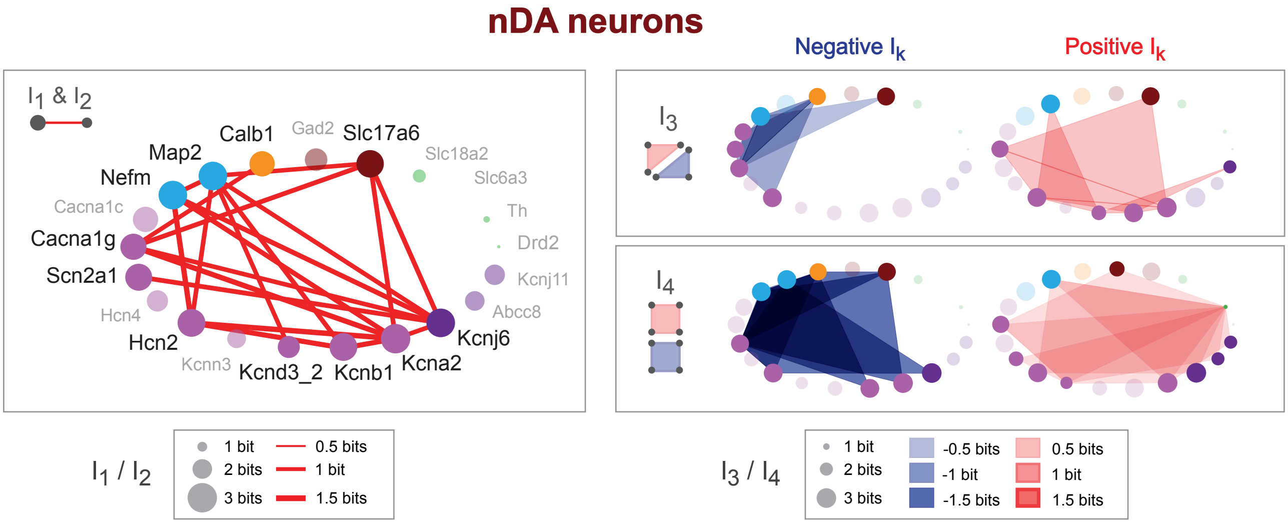

Supplementary Fig. 7. Gene modules in nDA neurons. scaffold representations of the most significant I_k values shared by pairs (I_2, 20 examples), triplets (I_3, 5) and quadruplets (I_4, 5) of genes in nDA neurons. Circle diameters are scaled according to entropy value (I_1). The red shapes indicate positive I_k shared by genes while the blue shapes correspond to negative I_k.

a

Positive I_K-sharing genes	Documented variation in midbrain DA neurons
***Slc6a3* / DAT**	Stronger expression in SNc than in VTA<br>*Poulin et al. (2014)*<br>*Figure 2 from Anderegg et al. (2015)*<br>*Reyes et al. (2013)*
***Kcnj6* / GIRK2**	Stronger expression in SNc than in VTA<br>*Poulin et al. (2014)*<br>*Figure 2 from Anderegg et al. (2015)*<br>*Reyes et al. (2012)*
***Kcnn3* / SK3**	Stronger SK3 labeling in SNc than in VTA<br>*Wolfart et al. (2001)*

Potential regulator	Documented variation in midbrain DA neurons
Pitx3	Stronger expression in SNc than in VTA<br>*Poulin et al. (2014)*<br>*Figure 2 from Anderegg et al. (2015)*

b

Negative I_K-sharing genes	Documented heterogeneity in midbrain DA neurons
***Calb1* / CB**	CB**+** in VTA and SNc subpopulations<br>*Damier et al. (1999)*<br>*González-Hernández et al. (2000)*
***Drd2* / D2R**	D2R- subpopulations in VTA<br>*Lammel et al. (2008)*<br>*Margolis et al. (2008)*
***Gad2* / GAD65**	GAD65**+** cells in VTA and SNc subpopulations<br>*González-Hernández et al. (2001)*
Abcc8* / SUR1**<br>Kcnj11* / Kir6.2**	Higher mRNA expression in SNc than in VTA<br>*Liss et al. (2005)*
***Cacna1c* / Cav1.2**	Higher Cav1.2 expression in lateral than in medial SNc. Differential expression of L-type channels in VTA and SNc<br>*Takada et al. (2001)*<br>*Philippart et al. (2016)*
***Cacna1g* / Cav3.1**	Larger T-type current density in CB- SNc cells<br>*Evans et al. (2017)*

Supplementary Fig. 8. Bibliographical support of the mutual information results obtained in the present study. a, some of the positive I_K-sharing genes (*Slc6a3, Kcnj6, Kcnn3*) have been reported to show strong variations in expression levels across midbrain DA neurons (using PCR, immunohistochemistry and/or electrophysiological recordings). Moreover, a recent study reporting variations in *Slc6a3* and *Kcnj6* also reported parallel variations in *Pitx3* expression, consistent with the proposed regulatory control of positive I_K-sharing genes by this transcription factor. **b**, negative I_K-sharing genes have heterogeneous profiles of expression in midbrain DA neurons. The table shows 7 genes sharing strong negative I_K and the list of articles reporting their heterogeneous expression (measured using PCR, immunohistochemistry or electrophysiological recordings).

Supplementary table S1: List of genes-TaqMan assays

Gene symbol	Protein	Official full name (ncbi)	Panther Molecular Function	Category	Criteria for inclussion	References	TaqMan assay ID/ primers and probe sequence	NCBI Ref Seq	Variants-isoforms	Amplicon length (bp)	Efficiency in multiplex (%)
Cacna1c	Cav1.2	Voltage-dependent L-type calcium channel	Voltage-gated calcium channel activity	Voltage-gated ion channel	DA neuron electric activity	*Chan et al., 2007; Dufour et al., 2014*	Mm01188822_m1	NM_001159533.2; NM_001159534.2; NM_001159535.2; NM_001255997.2; NM_001255998.2; NM_001255999.2; NM_001256000.2 ; NM_001256001.2; NM_001256002.2; NM_001290335.1; NM_009781.4	11	65	90.6
Cacna1d	Cav1.3	Voltage-dependent L-type calcium channel	Voltage-gated calcium channel activity	Voltage-gated ion channel	DA neuron electric activity	*Chan et al., 2007; Dufour et al., 2014*	Mm01209919_m1	NM_001083616.1; NM_028981.2	2	76	91.8
Cacna1g	Cav3.1	Voltage-dependent T-type calcium channel	Voltage-gated sodium channel activity	Voltage-gated ion channel	DA neuron electric activity	*Dufour et al., 2014; Poetschke et al., 2015*	Mm00486572_m1	NM_001112813.2 ; NM_001177888.1; NM_001177890.1; NM_009783.3	4	85	98.6
Hcn2	HCN2	Hyperpolarization activated sodium/potassium channel	Voltage-gated potassium channel activity	Voltage-gated ion channel	DA neuron electric activity	*Neuhoff et al., 2002; Dufour et al.,2014*	Mm00468538_m1	NM_008226.2	1	64	95.2
Hcn4	HCN4	Hyperpolarization activated sodium/potassium channel	Voltage-gated potassium channel activity	Voltage-gated ion channel	DA neuron electric activity	*Neuhoff et al., 2002; Dufour et al.,2014*	Mm01176086_m1	NM_001081192.1	1	70	99.1
Scn2a1	Nav1.2	Sodium channel, voltage-gated, type II, alpha 1	Voltage-gated sodium channel activity	Voltage-gated ion channel	DA neuron electric activity	*Seutin and Engel 2010; Ding et al., 2011*	Mm01270359_m1	NM_001099298.2	1	62	93.7
Scn5a	Nav1.5	Sodium channel, voltage-gated, type V, alpha	Voltage-gated sodium channel activity	Voltage-gated ion channel	Cardiac-cell electric activity	*Marionneau et al., 2005*	Mm01342518_m1	NM_001253860.1; NM_021544.4	2	59	99.7
Scn8a	Nav1.6	Sodium channel, voltage-gated, type VIII, alpha	Voltage-gated sodium channel activity	Voltage-gated ion channel	DA neuron electric activity	*Seutin and Engel 2010; Ding et al., 2011*	Mm00488110_m1	NM_001077499.2; NM_011323.3	2	68	94.1
Kcna2	Kv1.2	Voltage activated potassium channel	Voltage-gated potassium channel activity	Voltage-gated ion channel	DA neuron electric activity	*Ding et al., 2001; Fulton et al., 2011*	Mm00434584_s1	NM_008417.5	1	85	95.6
Kcnb1	Kv2.1	Voltage activated potassium channel	Voltage-gated potassium channel activity	Voltage-gated ion channel	DA neuron electric activity	*Ding et al., 2001; Dufour et al., 2014*	Mm00492791_m1	NM_008420.4	1	73	96.1
Kcnd2	Kv4.2	Voltage activated potassium channel	Voltage-gated potassium channel activity	Voltage-gated ion channel	DA neuron electric activity	*Ding et al., 2011*	Mm01161732_m1	NM_019697.3	1	66	97.8
Kcnd3	Kv4.3_2 (isoform 2, shorter)	Voltage activated potassium channel	Voltage-gated potassium channel activity	Voltage-gated ion channel	DA neuron electric activity	*Liss et al., 2001*	Mm00498260_m1	NM_019931.1	1 (isoform 2)	84	98.6
	Kv4.3_1 (isoform 1, longer)	Voltage activated potassium channel	Voltage-gated potassium channel activity	Voltage-gated ion channel	DA neuron electric activity	*Liss et al., 2001*	Mm01302125_m1	NM_001039347.1	1(isoform 1)	78	99.4

Kcnip3	KChip3	Kv channel interacting protein 3, calsenilin	Molecular function unclassified	Ion channel regulatory subunit	DA neuron electric activity	*An et al., 2000; Liss et al., 2001*	Mm01339774_m1	NM_001111331.1; NM_001291005.1; NM_019789.4	3	98	97.6
Kcnj11	Kir6.2	Inwardly rectifying K channel subunit (ATP-sensitive potassium (K-ATP) channels subunit)	Ligand-gated ion channel activity	ATP-activated inward potassium channel	DA neuron electric activity	*Liss et al., 1999*	Mm00440050_s1	NM_001204411.1; NM_010602.3	2	129	91.9
Abcc8	SUR1	Sulphonylurea receptor (ATP-sensitive potassium (K-ATP) channels subunit)	ATPase activity, coupled to transmembrane movement of substances/ transmembrane transporter activity	Ion channel regulatory subunit	DA neuron electric activity	*Liss et al., 1999*	Mm00803450_m1	NM_011510.3	1	77	94.7
Abcc9	SUR2B	Sulphonylurea receptor (ATP-sensitive potassium (K-ATP) channels subunit)	ATPase activity, coupled to transmembrane movement of substances/ transmembrane transporter activity	Ion channel regulatory subunit	DA neuron electric activity	*Liss et al., 1999*	Mm00441622_m1	NM_001044720.1 ; NM_011511.2; NM_021041.2; NM_021042.2 ; NM_001310143.1	5	75	92.5
Kcnj5	GIRK4	G protein-activated inward rectifier potassium channel 4 (potassium inwardly-rectifying channel, subfamily J, member 5)	Ligand-gated ion channel activity	G-protein activated inward rectifier potassium channel	Cardiac cell electric activity	*Marionneau et al., 2005*	Mm01175829_m1	NM_010605.4	1	83	95.9
Kcnj6	GIRK2_a, isoform a (b and 1)	G-protein-gated inwardly rectifying K+ channel	Ligand-gated ion channel activity	G-protein activated inward rectifier potassium channel	DA neuron electric activity	*Liss et al., 1999*	Mm01215648_m1	NM_001025584.2; NM_001025585.2; NM_010606.2	3 (isoforms a, b, 1)	97	99.5
	GIRK2_c, isoform c	G-protein-gated inwardly rectifying K+ channel	Ligand-gated ion channel activity	G-protein activated inward rectifier potassium channel	DA neuron electric activity	*Inanobe et al., 1999*	Mm01215646_m1	NM_001025590.1	1 (isoform c)	77	
Kcnn3	SK3	Small conductance calcium-activated potassium channel protein 3	Cation channel activity/ calmodulin binding	Calcium-activated potassium channel	DA neuron electric activity	*Wolfart et al., 2001; Deignan et al., 2012; Dufour et al., 2014*	Mm00446516_m1	NM_080466.2	1	71	96.5
Calb1	CB	Calbindin	Calcium ion binding	Calcium-binding protein	DA heterogeneity (VTA/SNc)	*Damier et al., 1999; Neuhoff et al., 2002*	Mm00486647_m1	NM_009788.4	1	78	93.7
Pvalb	PV	Parvalbumin	Calcium ion binding/ calmodulin binding	Calcium-binding protein	Interneuron marker	*Mercer et al., 2007*	Mm00443100_m1	NM_013645.3	1	77	94.4
Gfap	GFAP	Glial fibrillary acidic protein	Structural constituent of cytoskeleton	Structural (cytoskeleton) protein	Glia-specific marker		Mm01253033_m1	NM_001131020.1; NM_010277.3	2	75	99.0
Aldh1l1	FDH	Aldehyde dehydrogenase 1 family, member L1	Oxidoreductase activity	Enzyme	Glia-specific marker	*Cahoy et al., 2008; Pfrieger and Slezak 2012 (review);*	Mm03048957_m1	NM_027406.1	1	73	90.2
Th	TH	Tyrosine 3-monooxygenase (tyrosine hidroxylase)	Molecular function unclassified	Enzyme	DA metabolism		Mm00447546_m1	NM_009377.1	1	65	91.2

Slc6a3	DAT	Dopamine transporter	Cation transmembrane transporter activity/ neurotransmitter transporter activity	Dopamine transporter	DA metabolism/transport		Mm00438388_m1	NM_010020.3	1	74	97.8
Slc18a2	VMAT2	Synaptic vesicular amine transporter	Amino acid transmembrane transporter activity	Vesicular monoamine transporter	DA metabolism/transport		Mm00553058_m1	NM_172523.3	1	58	97.4
Drd2	D2R	Dopamine receptor 2	G-protein coupled receptor activity/adenylate cyclase activity/signal transducer activity	G-coupled receptor	DA signaling		Mm00438545_m1	NM_010077.2	1	62	96.2
Slc17a6	VGLUT2	Vesicular glutamate transporter 2	Amino acid transmembrane transporter activity	Vesicular glutamate transporter	Glutamate metabolism/transport	*Yamaguchi et al., 2013; Morales et al., 2014*	Mm00499874_m1	NM_080853.3	1	82	99.7
Gad1	GAD67	Glutamate decarboxylase 1	Carboxy-lyase activity	Enzyme	GABA metabolism	*Liss et al., 1999*	forward primer AGAGAAGAGTTTGAGATGGTTTTCG; reverse primer CTCGAAGGCTTTGTGGAATGTA; probe ATGGTGAGCCTGAGCAC	NM_008077.4	1	82	100.4
Gad2	GAD65	Glutamate decarboxylase 2	Carboxy-lyase activity	Enzyme	GABA metabolism	*Liss et al., 2005*	Mm00484623_m1	NM_008078.2	1	99	100.3
Chat	CHAT	Choline acetyltransferase	Acetyltransferase activity	Enzyme	Ach metabolism	*Mercer et al., 2007*	Mm01221882_m1	NM_009891.2	1	67	96.9
Penk	ENK	Proenkephalin-A precursor	Neuropeptide hormone activity	Neuropeptide	Interneuron marker	*Mercer et al., 2007*	Mm01212875_m1	NM_001002927.2	1	57	97.1
Ncam2	NCAM2	Neural cell adhesion molecule isoform 2	Molecular function unclassified	Structural (membrane) protein	Neuronal structure		Mm01344613_m1	NM_001113208.1; NM_010954.4	2	80	96.2
Map2	MAP2	Microtubule asociated protein 2	Microtubule binding	Structural (cytoskeleton) protein	Neuronal (dendritic) structure		Mm00485231_m1	NM_001039934.1; NM_008632.2	2	118	91.8
Nefm	NEF3	Neurofilament medium polypeptide	Structural constituent of cytoskeleton	Structural (cytoskeleton) protein	Neuronal (axonal) structure	*Weng et al., 2010*	Mm00456201_m1	NM_008691.2	1	84	99.9
Creb1	CREB	Cyclic AMP-responsive element-binding protein	Molecular function unclassified	Transcription factor	Neuronal activity	*Greer et al., 2008; Okuno et al., 2011*	Mm00501607_m1	NM_001037726.1; NM_009952.2; NM_133828.2	3	106	98.2
Fos	C-FOS	FBJ osteosarcoma oncogene	Molecular function unclassified	Transcription factor	Neuronal activity	*Greer et al., 2008; Okuno et al., 2011*	Mm00487425_m1	NM_010234.2	1	59	98.7
Bdnf	BDNF	Brain-derived neurotrophic factor	Growth factor activity/ neurotrophin receptor binding	Secretor factor, neurotrophin	Neuronal activity	*Greer et al., 2008; Okuno et al., 2011*	Mm04230607_s1	NM_001048139.1; NM_001048141.1; NM_001048142.1; NM_001285416.1; NM_001285417.1; NM_001285418.1; NM_001285419.1; NM_001285420.1; NM_001285421.1; NM_007540.4; NM_001285422.1;	11	93	95.9
Hprt	HGPRT	hypoxanthine guanine phosphoribosyl transferase	Binding/ transferase activity, transferring glycosyl groups	Enzyme	Housekeeping gene	*Marionneau et al., 2005; Santos et al. 2008*	Mm00446968_m1	NM_013556.2	1	65	91.1

Tbp	TBP	TATA-box-binding protein	Sequence-specific DNA binding transcription factor activity	Transcription factor	Housekeeping gene	*Santos et al. 2008; Swijsen et al. 2012*	Mm00446971_m1	NM_013684.3	1	93	89.4
Tbx3	TBX3	T-box transcription factor 3	Sequence-specific DNA binding transcription factor activity	Transcription factor	Sino-atrial node cell specific marker	*Mangoni and Nargeot 2008*	Mm01195726_m1	NM_011535.3; NM_198052.2	2	65	91.4
